# An Adaptive Ellipse Distance Density Peak Fuzzy Clustering Algorithm Based on the Multi-target Traffic Radar

**DOI:** 10.3390/s20174920

**Published:** 2020-08-31

**Authors:** Lin Cao, Xinyi Zhang, Tao Wang, Kangning Du, Chong Fu

**Affiliations:** 1 Key Laboratory of the Ministry of Education for Optoelectronic Measurement Technology and Instrument, Beijing Information Science and Technology University, Beijing 100192, China; charlin@bistu.edu.cn (L.C.); zhangxinyi0215@163.com (X.Z.); kangningdu@bistu.edu.cn (K.D.); 2 School of Information and Communication Engineering, Beijing Information Science and Technology University, Beijing 100101, China; 3 School of Computer Science and Engineering, Northeastern University, Shenyang 110004, China; fuchong@mail.neu.edu.cn

**Keywords:** multi-target traffic radar scene, adaptive ellipse distance, decision diagram, density peak point, fuzzy clustering

## Abstract

In the multi-target traffic radar scene, the clustering accuracy between vehicles with close driving distance is relatively low. In response to this problem, this paper proposes a new clustering algorithm, namely an adaptive ellipse distance density peak fuzzy (AEDDPF) clustering algorithm. Firstly, the Euclidean distance is replaced by adaptive ellipse distance, which can more accurately describe the structure of data obtained by radar measurement vehicles. Secondly, the adaptive exponential function curve is introduced in the decision graph of the fast density peak search algorithm to accurately select the density peak point, and the initialization of the AEDDPF algorithm is completed. Finally, the membership matrix and the clustering center are calculated through successive iterations to obtain the clustering result.The time complexity of the AEDDPF algorithm is analyzed. Compared with the density-based spatial clustering of applications with noise (DBSCAN), *k*-means, fuzzy c-means (FCM), Gustafson-Kessel (GK), and adaptive Euclidean distance density peak fuzzy (Euclid-ADDPF) algorithms, the AEDDPF algorithm has higher clustering accuracy for real measurement data sets in certain scenarios. The experimental results also prove that the proposed algorithm has a better clustering effect in some close-range vehicle scene applications. The generalization ability of the proposed AEDDPF algorithm applied to other types of data is also analyzed.

## 1. Introduction

In the intelligent transportation system, multi-target traffic radar is used as a road traffic assistance tool to obtain information such as the speed and distance of vehicles in multiple lanes of the radar irradiation area, which is widely used in road speeding bayonet snapshots and traffic Information monitoring [1,2]. The use of road vehicle detection algorithms can help reduce the occurrence of traffic accidents and effectively promote the development of intelligent transportation [3,4]. Currently, there are many image-based multi-target detection algorithms, but the performance of the algorithm is greatly restricted by the environment [5,6]. More and more researchers have begun to use radar to solve the problem of multi-target vehicle detection in the transportation field because the signals emitted by the radar can adapt to the changing environment.

As a sensor that can work around the clock, multi-target radar can overcome the deficiencies of the camera in the climate and has the advantages of high-speed measurement accuracy, convenient maintenance, low installation cost, and high stability [7,8]. Multi-target radar can record the number of vehicles in different lanes per unit time to achieve traffic flow statistics in lanes. At the same time, multi-target radar can accurately detect the speed information of vehicles in different lanes, determine whether the vehicle is over-speeding, and upload the over speed violation information to the relevant law enforcement departments [9]. Besides, the multi-target radar determines the congestion of vehicles on the current road section based on the information detected at important road sections such as traffic light intersections. Multi-target radar has become one of the most used road equipment in intelligent transportation systems and is widely applied to vehicle flow statistics, speeding vehicle monitoring, and lane congestion information judgment [10].

**Figure 1:**
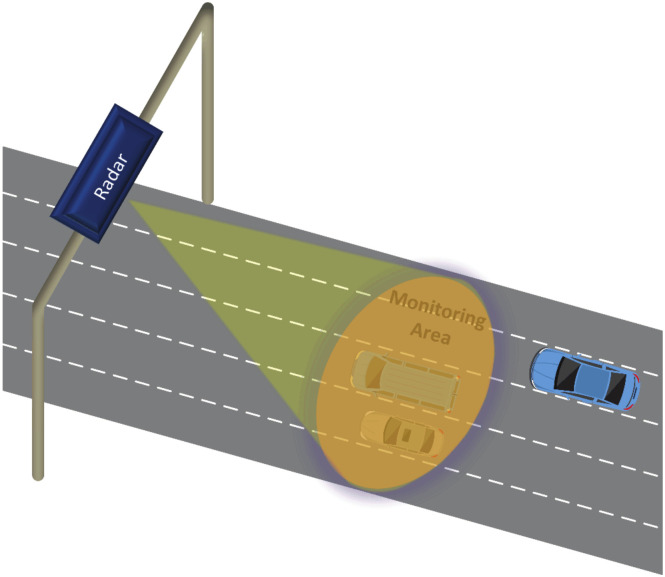
Schematic diagram of top-mounted multi-target radar.

**Figure 2:**
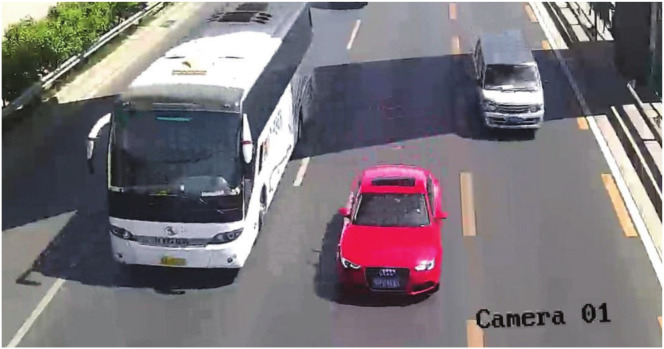
Actual radar scene.

Multi-target traffic radar is a very important branch in intelligent transportation, which can provide many necessary vehicle target information [11]. This information can effectively guarantee traffic safety and avoid traffic accidents. The multi-target traffic radar is installed directly above multiple lanes or beside the outermost lane. Top-mounted and side-mounted are common installation methods for multi-target traffic radar [12]. As shown in Figure 1, the multi-target traffic radar in this scenario uses a top-mounted installation method. When multiple vehicles enter the radar detection area, the multi-target traffic radar can detect multiple vehicle targets at the same time and collect reflected signals from the vehicle targets. Afterward, the multi-target traffic radar processes these signals to obtain the detection points of the target vehicles, thus completing the detection of the vehicles in the radar irradiation area. As shown in Figure 2, this paper uses the real scene of a four-lane city road.

In real road conditions, it is often the case that multiple vehicles travel close to each other when traveling at a horizontal distance or a vertical distance. This makes the detection points generated after radar signal processing interleaved and difficult to distinguish and increases the difficulty of subsequent information processing to a certain extent [13]. Besides, the acquisition of important traffic data such as distance measurement, speed measurement, and flow statistics also needs to be carried out smoothly under the premise of correct vehicle detection information [14]. Therefore, this paper intends to solve the above problems, and the radar detection points are processed by the algorithm proposed in this paper. The algorithm proposed in this paper can more accurately distinguish the vehicle targets in the current scene and obtain the number and location information of the vehicle targets, which is convenient for subsequent data processing.

In recent years, many classic clustering algorithms are used in the field of traffic radar, including the density-based spatial clustering of applications with noise (DBSCAN) algorithm, the *k*-mean clustering algorithm, and the fuzzy *c*-means (FCM) clustering algorithm and so on [15]. As a density-based spatial clustering algorithm, the DBSCAN algorithm has a poor clustering effect when the distance between different data categories is close or even intersects [16]. The *k*-means algorithm is a typical distance-based algorithm, which uses distance as a similarity evaluation index, but the *k*-means algorithm itself has low sensitivity to changes in data density, and the choice of *k* value is not easy to grasp [17]. As an improvement of *k*-means, FCM is an unsupervised classification algorithm, and its clustering results can better reflect the class structure of the data itself and have better interpretability [18]. However, the FCM algorithm needs to give the number of clusters in advance, and the clustering effect on data with a super ellipsoid shape is not good [19]. In the Gustafson-Kessel (GK) clustering algorithm, Gustafson and Kessel replace the Euclidean distance in the FCM algorithm with Markov distance, so that the algorithm can cluster non-spherical data [20]. As a fuzzy clustering algorithm, GK has the limitation that it needs to initialize the clustering center and select the optimal number of clusters. Different parameter selection and initialization methods in the GK algorithm may lead to different classification results, and even lead to incorrect classification [21].

There are many new achievements in the field of clustering algorithms, and there are many improvements to existing algorithms. Jiahu Qin and Weiming Fu proposed a distributed *k*-means++ algorithm, which can achieve faster convergence speed and results with global optimality [22]. Miin-Shen Yang and Yessica Nataliani improved the FCM algorithm and proposed the feature-reduction FCM (FRFCM) algorithm, which can automatically calculate the weight of a single feature while reducing these unrelated feature components [23]. Liu, Xinwang, Zhu, Xinzhong, and others directly solved the case where the rows and columns of some basic kernel matrices did not exist by directly performing multi-core clustering in the presence of incomplete kernel matrices [24]. Deep Embedded Clustering (DEC) is a clustering method based on deep learning, which uses deep neural networks to simultaneously learn feature representations and cluster assignments [25]. Yang J. and Parikh D. proposed a cyclic recurrent for joint unsupervised learning of deep representation and image clustering. The clustering performance of this method is good on many image datasets [26]. In 2014, Alex Rodrigblez and Alessandro jointly proposed the clustering by fast search and find of density peaks (CFSFDP) algorithm [27]. The principle of this algorithm is simple, only one parameter is needed as input, and it does not need too many iterations. However, the CFSFDP algorithm also has shortcomings. The algorithm uses the Euclidean distance to calculate the distance between data points, which has limitations in describing the shape of the cluster. Secondly, the algorithm needs to set a threshold as the parameter measurement center point when looking for the density peak point. This threshold is fixed and difficult to set in practical applications [28].

According to the data characteristics of vehicle targets in the close-range vehicle scene, this paper proposes an adaptive ellipse distance density peak fuzzy (AEDDPF) clustering algorithm. This algorithm solves the problem that the existing algorithms in the above scenarios have a poor effect on vehicle clustering. Firstly, to accurately describe the ellipse structure of the radar data cluster in the neighborhood vehicle scene, the algorithm uses an adaptive ellipse distance to calculate the similarity between points. Secondly, an adaptive exponential function curve is introduced in the decision graph of the fast density peak search algorithm to select the density peak point accurately, and the initialization of the AEDDPF algorithm is completed. Finally, the membership matrix and clustering center of the AEDDPF algorithm are continuously iterated to obtain the clustering result.

In summary, the main contributions of this paper are as follows:

- The initial cluster center points and the number of center points are effectively obtained. To initialize the AEDDPF clustering algorithm, the adaptive ellipse distance is used to accurately describe the ellipse distribution characteristics of the data cluster in the current radar scene. Then, an adaptive exponential function curve is introduced in the decision graph of the fast density peak search algorithm to select the density peak point accurately.
- An AEDDPF clustering algorithm is proposed. As a fuzzy clustering algorithm, the algorithm does not need to set the number of initial centers in advance, or randomly generate initial centers. On the contrary, the algorithm only needs to find the density peak point in the decision graph through the exponential function, and then accurately find the initial clustering center. The clustering center after initialization is very close to the actual target clustering center, thereby reducing the number of subsequent iterations.

The organization structure of this article is as follows. In Section 2, the GK and CFSFDP algorithms related to the AEDDPF clustering algorithm are reviewed. In Section 3, the AEDDPF clustering algorithm is described in detail, and the time complexity of the algorithm is analyzed. In Section 4, the process of acquiring and processing radar data is introduced. Then, the experimental of several real urban road scenes are described, the performance of several algorithms is compared with the AEDDPF algorithm, and the effectiveness of the algorithm is verified. After that, the generalization ability of the proposed AEDDPF clustering algorithm applied to other types of data is analyzed. In Section 5, the conclusion is given.

## 2. Related Algorithms

### 2.1. CFSFDP Algorithm

The CFSFDP algorithm is also known as the density peak algorithm. At the beginning of the discovery, there was a warm response in the academic community, and many scientific researchers were also attracted to study the algorithm. The core of the algorithm is the description of the clustering center. The algorithm believes that the clustering center has the following two characteristics. Firstly, the neighborhood density points with low local density surround the cluster center, which is the peak density point. Secondly, the distance between any clustering center and the data points with a higher density is longer. According to the above two characteristics of the cluster center, for a data set, the cluster center is surrounded by some data points with low local density. The distance between these local low-density data points and other local high-density data points is large.

For any point $q_{k}$ in the data set $Q={\left\{q_{k}\right\}}_{k=1}^{N}$, CFSFDP calculates the local density $\omega_{k}$ of the data point $q_{k}$, the minimum distance $\varphi_{k}$ between the data point $q_{k}$ and the high-density point, and the distance between the data point $q_{k}$ and other data points. It is worth noting that the truncated kernel and Gaussian kernel are two commonly used methods of local density calculation.The truncated kernel are used to calculate the local density $\omega_{k}$ of the data points $q_{k}$. The local density $\omega_{k}$ can be expressed as

(1) $$\omega_{k}=\sum_{p-1}^{N}\chi \left(d_{kp}-d_{c}\right),$$

where *N* represents the number of data points, $d_{kp}$ is the distance between the data point $q_{k}$ and $q_{p}$. The most used distance measurement method here is the Euclidean distance. $d_{c}$ is the truncation distance, which is usually the distance threshold specified in advance, and can be calculated according to the ratio of the total number of points about the data point to the total number of data points in the data set. χ is a truncated core, when x<0,χ(x)=1, otherwise χ(x)=0. The definition of Equation (1) shows that $\omega_{k}$ is the number of data points distributed in the $d_{c}$ neighborhood of $q_{k}$.

In addition to using the truncated kernel in Equation (1) to calculate the local density calculation, the Gaussian kernel can also be used to calculate the point density, which is defined as follows:

(2) $$\omega_{k}=\sum_{p\neq k}e^{-{\left(\frac{d_{kp}}{d_{c}}\right)}^{2}}.$$

By observing Equation (1) and (2), the local density obtained by truncating the kernel is a discrete value, while the local density value obtained by the Gaussian-kernel is a continuous value. The discrete value obtained by truncating the kernel will cause different data points to have the same local density value, which is not conducive to the subsequent classification work. Therefore, it is more reasonable to use the Gaussian kernel to calculate the local density. $\varphi_{k}$ is measured by calculating the minimum distance between the point $q_{k}$ and other data points with higher local density, which is defined as

(3) $$\varphi_{k}=\min_{k:\omega_{k}<w_{p}}\left(d_{kp}\right).$$

**Figure 3:**
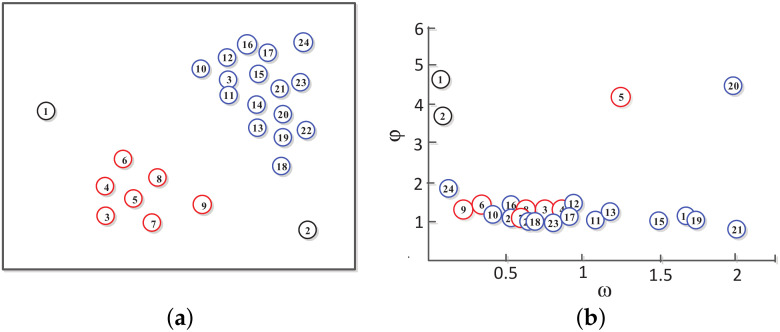
Two-dimensional data point model diagram: (**a**) Data distribution; (**b**) Decision diagram.

Based on the local density ω and minimum distance φ, CFSFDP obtains a decision graph and divides the data points into density peak points, core points, and outlier noise points. As shown in Figure 3a, the discrete points of the data set are distributed on a two-dimensional plane. As shown in Figure 3b, the points in the data set are arranged in such a manner that the density value decreases. Points 5 and 20 are prominently located in the decision graph, and are distributed in the upper right part of the graph, and have large ω and φ values. There are no data points with a higher density than these two points in the larger neighborhood, so these two points meet the two conditions of the cluster center, which are the peak density points. Although point 21 has a very high ω value, but the φ value is very small, which is distributed in the lower right corner of Figure 3b. This indicates that there is a point with higher density in the neighborhood of point 21, so it is a non-peak point, which does not meet the condition of the cluster center. Points 1 and 2 have higher φ values, but the ω values are very low, both distributed in the upper left corner of the graph, indicating that they are outliers.

The CFSFDP algorithm can calculate the local density ω and minimum distance φ for each data point in the data set, and use the data points with larger ω and φ values as the clustering center. After that, the CFSFDP algorithm assigns the remaining data points to the class closest to each cluster center. The user can manually select the cluster center point in the decision graph or can set a threshold γ=φω. The CFSFDP algorithm treats the points greater than the threshold γ as the clustering center, and then assigns the remaining points to the clusters in the nearest neighboring area with higher density in turn.

### 2.2. GK Clustering Algorithm

The GK algorithm is an improved FCM algorithm and belongs to one of the most widely used algorithms in fuzzy clustering algorithms [29]. Therefore, the principles of the GK algorithm and the FCM algorithm are similar, both of which are based on the similarity between sample points and categories to calculate the membership of a certain category. Among them, the degree of membership indicates the degree of membership of samples belonging to a certain category. In other words, like FCM, the GK algorithm allows data points to belong to two or more classes at the same time, and the degree to which each data point belongs to a class is limited by the degree of membership, thereby achieving the purpose of data classification. In this section, the FCM clustering process is first introduced, and then the GK algorithm is introduced by analogy.

Let the set ${\left\{q_{k}\right\}}_{k=1}^{N}\subset R^{S}$ represent a given sample set. For the radar data set, the sample set is the coordinate value of each vector point in the data set. *S* is the dimension of the sample space. *N* is the number of samples. The collection is now divided into c(2≤c≤N) categories. $Z=\left(z_{1},z_{2},\cdots ,z_{c}\right)$ is a consists of *c* cluster center vectors. The FCM algorithm uses the number of elements in the set *Z* to represent the fuzzy *c* division of the data set.

The fuzzy membership matrix $B={\left[b_{rk}\right]}_{c\times N}$ of FCM meets the following constraints

(4) $$\sum_{r=1}^{c}b_{rk}=1,1\leq k\leq N,1\leq r\leq c,$$

(5) $$b_{rk}\in \left[0,1\right],1\leq k\leq N,1\leq r\leq c.$$

The objective function of the FCM is defined as follows:

(6) $$J_{FCM}\left(B,Z\right)=\sum_{r=1}^{c}\sum_{k=1}^{N}b_{rk}^{m}d_{kr}^{2}.$$

In Equation (6), m>1 is called the fuzzy index. $d_{kr}=∥q_{k}-z_{r}∥$ represents the distance description from the data point $q_{k}$ to $z_{r}$. There are many ways to describe the distance here, and the most used is the Euclidean distance.

To minimize the objective function $J_{FCM}$, the Lagrange multiplier method is used to find the limit value.

(7) $$z_{r}=\frac{\sum_{k=1}^{N}b_{rk}^{m}q_{k}}{\sum_{k=1}^{N}b_{rk}^{m}},$$

(8) $$b_{rk}=\frac{1}{\sum_{l=1}^{c}{\left(\frac{d_{kr}}{d_{rl}}\right)}^{\frac{2}{m-1}}}.$$

The FCM algorithm minimizes the objective Equation (6) by iteratively updating the clustering center and membership matrix. When the membership matrix obtained in two iterations meets certain conditions, the iteration ends.

Gustafson and Kessel and others changed the Euclidean distance to the Mahalanobis distance measurement method and proposed the GK fuzzy clustering algorithm. The shortcoming of Markov distance is that it cannot be directly used in the distance measurement of the clustering algorithm. Using Markov distance directly as a distance measurement may make samples belonging to different categories have the same degree of membership, thereby affecting the accuracy of clustering results.

The objective function of the GK algorithm is

(9) $$J_{GK}\left(B,Z\right)=\sum_{r=1}^{c}\sum_{k=1}^{N}{\left(b_{rk}\right)}^{m}\left[\left(q_{k}-z_{r}\right)A_{r}\left(q_{k}-z_{r}\right)\right].$$

$A_{r}$ is a positive definite matrix. The principles of GK and FCM clustering algorithms are similar. The algorithm also makes the objective function reach the minimum value through iteration and stops iterating when the convergence criterion is met.

Compared with the limitation of FCM for describing the shape of point cluster clustering, GK clustering is an improved fuzzy partitioning scheme. In practical applications, the shortcomings of GK are also very prominent. It is very sensitive to initializing cluster centers and needs to select the best number of clusters. Therefore, the improvement of the initialization scheme of the GK algorithm to make it more accurate to initialize the cluster center and the correct number of clusters will greatly improve the stability and accuracy of the algorithm.

## 3. AEDDPF Clustering Algorithm

The use of secondary clustering to correct the clustering results is the characteristic of the AEDDPF algorithm proposed in this paper. The algorithm uses adaptive ellipse distance to process data. To obtain the center coordinates of each cluster and the number of point clusters in the data, an exponential function curve is adaptively drawn in the decision diagram to complete the rapid selection of the density peak point. By constructing the objective function, the algorithm continuously iterates the membership matrix and clustering center, and finally obtains the clustering result. This section will detail the construction process of the AEDDPF algorithm.

### 3.1. Initialization of AEDDPF Clustering Algorithm

The AEDDPF algorithm performs cluster analysis on the sample points in the two-dimensional radar dataset $Q={\left\{q_{k}\right\}}_{k=1}^{N}\subset R^{2}$. $I_{S}=\left\{1,2,\cdots ,N\right\}$ is the index set of data set *Q*. *N* is the total number of vectors in the point set. The data point coordinates are expressed as $q_{k}=\left(x_{k},y_{k}\right)$.

#### 3.1.1. Adaptive Ellipse Distance

This paper uses adaptive ellipse distance to process the data distance in the AEDDPF algorithm. In measuring the distance similarity between data points, the adaptive ellipse distance considers setting two variable parameters to adjust the similarity between data. If the value of the corresponding element in the data is closer and the element contributes more in the calculation, then this element should have a greater weight in the calculation. The distance similarity obtained after this processing can more accurately reflect the complex structural features of the data set. The calculation formula of the adaptive ellipse distance is as follows:

(10) $$dis_{k\ell }=\sqrt{\frac{{\left|y_{k}-y_{\ell }\right|}^{2}}{{\left(W_{a_{k}}\right)}^{2}}+\frac{{\left|x_{k}-x_{\ell }\right|}^{2}}{{\left(W_{b_{k}}\right)}^{2}}},$$

where $W_{a_{k}}$ and $W_{b_{k}}$ are the weight parameters of the ellipse long axis and the weight parameters of the ellipse semi-axis, respectively. These three parameters are specifically expressed as

(11) $$\left\{\begin{array}{c} W_{a_{k}}=\frac{W_{k\ell }}{W_{k\ell_{1}}}\\ W_{b_{k}}=\frac{W_{k\ell }}{W_{k\ell_{2}}}, \end{array}\right.$$

where $W_{k\ell }$ is the change factor and satisfies $W_{k\ell }=W_{k\ell_{1}}+W_{k\ell_{2}}$. $W_{{k\ell }_{1}}$ and $W_{{k\ell }_{2}}$ are expressed as

(12) $$\left\{\begin{array}{c} W_{k\ell_{1}}=e^{\frac{-{\left|x_{k}-x_{\ell }\right|}^{2}}{\alpha }}\\ W_{k\ell_{2}}=e^{\frac{-{\left|y_{k}-y_{\ell }\right|}^{2}}{\alpha }}. \end{array}\right.$$

Take α=1 in the experiment, which can better balance the horizontal and vertical coordinates of different data points. This balance is set according to the weight of each data point.

One advantage of the AEDDPF algorithm is the introduction of adaptive ellipse distance. This distance can reflect the different functions of each variable in the data and improve the clustering results. The adaptive ellipse distance assigns a variable weight value to each variable in the sample point, which can reflect the different functions of each variable in the data. Without changing the algorithm framework, the adaptive ellipse distance makes full use of the characteristics of the data itself to improve the accuracy of the data distance similarity description.

#### 3.1.2. Selecting the Initial Clustering Center Based on the Adaptive Exponential Function

To accurately select the initial clustering center, the AEDDPF algorithm will define the initial clustering center as follows:

- The initial cluster center point has a high local density and is surrounded by neighboring points with a relatively low local density.
- The distance between the cluster center point and other low-density neighborhood points is large and can be selected by an adaptive exponential curve of the over-density mean point.

For each data point $q_{k}$, only the local density $\omega_{k}$ of the point $q_{k}$ and the shortest distance $\varphi_{k}$ from the point $q_{k}$ to the point $q_{\ell }$ with higher local density need to be calculated. The Gaussian kernel obtains continuous values, so that different data points have different local density values, which is conducive to the subsequent classification work. Substituting the adaptive ellipse distance $dis_{k\ell }$ in Equation (10) into the Gaussian kernel can obtain the local density $\omega_{k}$. Their expression is defined as

(13) $$\begin{array}{cc} \omega_{k} & =\sum_{\ell =1}^{N}\exp\left(\frac{-dis_{k\ell }^{2}}{\eta }\right)\\ \; & =\sum_{\ell =1}^{N}\exp\left(-\frac{1}{\eta }\left(\frac{{\left|y_{k}-y_{\ell }\right|}^{2}}{{\left(W_{a_{k}}\right)}^{2}}+\frac{{\left|x_{k}-x_{\ell }\right|}^{2}}{{\left(W_{b_{k}}\right)}^{2}}\right)\right). \end{array}$$

The distance threshold η specified for the algorithm can usually be taken as the first 1% to 2% of the distance between all data points in ascending order. For each data point $q_{k}$, after calculating the local density $\omega_{k}$, the shortest distance $\varphi_{k}$ from the point $q_{k}$ to $q_{\ell }$ with higher local density and the distance $\varphi_{\ell }$ of $q_{\ell }$ need to be calculated. $\varphi_{k}$ and $\varphi_{\ell }$ are defined as

(14) $$\left\{\begin{array}{c} \varphi_{k}=\min_{k\in I_{S}}\left(dis_{k\ell }\right),I_{S^{\prime }}\neq ⌀\\ \varphi_{\ell }=\max_{\ell \in I_{S}}\left(dis_{k\ell }\right),I_{S^{\prime }}=⌀. \end{array}\right.$$

The expression of the index set in which the density values are arranged in descending order is shown in Equation (15).

(15) $$I_{S^{\prime }}=\left\{\ell \in I_{S}:\omega_{\ell }>\omega_{k}\right\}.$$

The local density $\omega_{k}$ and shortest distance $\varphi_{k}$ of all element points in the data set *Q* are calculated. Combining these two values results in a set of density points $H={\left\{\zeta_{k}\right\}}_{k=1}^{N}$, where the coordinates of the density points $\zeta_{k}$ are $\left(\omega_{k},\varphi_{k}\right)$. According to the coordinates of these density points, a two-dimensional plane decision map can be drawn.

The distance between the density peak point and the density point is relatively long, and at the same time has a large local density $\omega_{k}$ and the shortest distance $\varphi_{k}$. An exponential function curve of the mean density point is constructed, which is used to adaptively select the density peak point. The density point above the adaptive exponential function curve meets the two conditions of the density peak point, which is the density peak point. The construction process of the adaptive exponential function is given below.

The mean density point $\bar{\zeta }\left(\bar{\omega },\bar{\varphi }\right)$ is calculated from the mean value of all density points in the set of density points *H*. The calculation formula of the horizontal and vertical coordinates of the density mean point is

(16) $$\left\{\begin{array}{c} \bar{\omega }=\frac{1}{N}\sum_{k=1}^{N}\omega_{k}\\ \bar{\varphi }=\frac{1}{N}\sum_{k=1}^{N}\varphi_{k}. \end{array}\right.$$

The expression of the adaptive exponential function is

(17) $$f\left(\omega \right)=Ke^{\frac{1}{\omega }}.$$

The adaptive exponential function f(ω) is a compound function curve passing through the mean point of density $\bar{\zeta }\left(\bar{\omega },\bar{\varphi }\right)$, and the coefficient is $K=\bar{\varphi }e^{\frac{-1}{\bar{\omega }}}$.

When the distance value $\varphi_{k}$ of the density point is greater than the exponential function value $f(\omega_{k})$ of the vertical direction, the current density point is regarded as the density peak point. The corresponding point $q_{k}$ belongs to the element in the cluster center matrix *Z*, where c(2≤c≤N) is the number of clusters. The formula for selecting the initial cluster center is

(18) $$\left\{\begin{array}{c} q_{k}\in Z,\varphi_{k}>f\left(\omega_{k}\right)\\ q_{k}\notin Z,\;\mathrm{otherwise}\;. \end{array}\right.$$

Each data set can calculate a corresponding density mean point $\bar{\zeta }$. From this specific density mean point $\bar{\zeta }$, the corresponding *K* value of the Equation (17) can be obtained. Thus, the complete expression of the adaptive exponential function corresponding to the current data set is obtained. The adaptive exponential function obtained above changes with the distribution of the density points of the data set. In other words, the exponential function here can adaptively select the peak density point. The resulting density peak point is the initial cluster center point of the AEDDPF algorithm. So far, this section has completed the initialization part of the AEDDPF algorithm.

### 3.2. The Main Part of AEDDPF Algorithm

In the AEDDPF algorithm model, the initial clustering center set $Z=\left(z_{1},z_{2},\cdots ,z_{c}\right)$ of clustering is obtained after initialization. The data set *Q* is now divided into *c* categories. Let $b_{rk}$ be the membership of the m sample point to the *c*-type, and satisfy $b_{rk}\in \left[0,1\right],\sum_{r=1}^{c}b_{rk}=1,r=1,2,\cdots ,c$. The fuzzy membership matrix is expressed as

(19) $$B=\left[\begin{array}{cccc} b_{11} & b_{12} & \cdots & b_{1N}\\ b_{21} & b_{22} & \cdots & b_{2N}\\ \vdots & \vdots & \ddots & \vdots \\ b_{c1} & b_{c2} & \cdots & b_{cN}. \end{array}\right]$$

The objective function of the AEDDPF algorithm is defined as

(20) $$J_{AEDDPF}\left(B,Z\right)=\sum_{r=1}^{c}\sum_{k=1}^{N}{\left(b_{rk}\right)}^{m}D_{rk}^{2}\left(q_{k},z_{r}\right),$$

where $D_{rk}^{2}\left(q_{k},z_{r}\right)$ is the distance norm, which represents the distance between the sample $q_{r}$ and the cluster center of the category *r*, which is used to describe the shape of the current cluster.

(21) $$D_{rk}^{2}\left(q_{k},z_{r}\right)=\left(q_{k}-z_{r}\right)A_{r}\left(q_{k}-z_{r}\right),$$

where $A_{r}$ is a positive definite matrix, which is determined by the clustering covariance matrix $F_{r}$. The eigenvalues and eigenvectors of the clustering covariance matrix $F_{r}$ provide information about the shape of the cluster. The definitions of $F_{r}$ and $A_{r}$ are as follows:

(22) $$F_{r}=\frac{\sum_{k=1}^{N}{\left(b_{rk}\right)}^{m}{\left(q_{k}-z_{r}\right)}^{T}\left(q_{k}-z_{r}\right)}{\sum_{k=1}^{N}{\left(b_{rk}\right)}^{m}},$$

(23) $$A_{r}=\frac{{\left(\rho_{r}\det\left(F_{r}\right)\right)}^{\frac{1}{2}}}{F_{r}}.$$

For each category, $\rho_{r}$ is a constant. In the absence of prior knowledge, take $\rho_{r}=1$. m>1 is the fuzzy index. $\det\left(F_{r}\right)$ represents the determinant of the clustering covariance matrix $F_{r}$. To optimize the objective function in Equation (20), the Lagrange multiplier method is used to find the extreme value. Thus, the fuzzy membership $b_{rk}$ and clustering center $z_{r}$ corresponding to the objective function is obtained.

The specific solution process of fuzzy membership $b_{rk}$ and clustering center $z_{r}$ is as follows:

First, the constraint condition $\sum_{r=1}^{c}b_{rk}=1$ is brought into the objective function of Equation (20) by using the Lagrange multiplier method:

(24) $$L\left(b_{rk},z_{r},\tau_{k}\right)=\sum_{k=1}^{N}\left(\sum_{r=1}^{c}b_{rk}^{m}D_{rk}^{2}\left(q_{k},z_{r}\right)+\tau_{k}\left(1-\sum_{r=1}^{c}b_{rk}^{m}\right)\right),$$

where $\tau_{k}$ represents the Lagrange multiplier of constraint $\sum_{r=1}^{c}b_{rk}=1$. To calculate the extreme value of Equation (24), the partial derivatives of *L* with respect to membership $b_{rk}$ and clustering center $z_{r}$ need to be found separately. Then, the two partial derivatives of *L* above are set to zero. The expression is as follows:

(25) $$\left\{\begin{array}{c} \frac{\partial L}{\partial b_{rk}}=mD_{rk}^{2}\left(q_{k},z_{r}\right)b_{rk}^{m-1}-\lambda_{k}=0\\ \frac{\partial L}{\partial z_{r}}=-\sum_{k=1}^{N}2b_{rk}^{m}\left(q_{k}-z_{r}\right)=0. \end{array}\right.$$

According to Equation (25), the following expression can be obtained as

(26) $$\left\{\begin{array}{c} z_{r}=\frac{\sum_{k=1}^{N}b_{rk}^{m}q_{k}}{\sum_{k=1}^{N}b_{rk}^{m}}\\ b_{rk}=\frac{1}{{\left(\frac{D_{rk}^{2}\left(q_{k},z_{r}\right)}{D_{r1}^{2}\left(q_{1},z_{r}\right)}\right)}^{\frac{1}{m-1}}+{\left(\frac{D_{rk}^{2}\left(q_{k},z_{r}\right)}{D_{r2}^{2}\left(q_{2},z_{r}\right)}\right)}^{\frac{1}{m-1}}+\cdots +{\left(\frac{D_{rk}^{2}\left(q_{k},z_{r}\right)}{D_{rc}^{2}\left(q_{c},z_{r}\right)}\right)}^{\frac{1}{m-1}}}. \end{array}\right.$$

Equation (26) shows that fuzzy membership $b_{rk}$ and the clustering center $z_{r}$ are related to each other. Before iteratively calculating the AEDDPF algorithm, the initial value $b_{rk}$ is needed to set. During the iterative process, the objective function of AEDDPF is constantly changing, and it gradually tends to a stable value. The AEDDPF algorithm stops iterating after meeting the convergence criteria and outputs the result.

The implementation framework of the AEDDPF algorithm (Algorithm 1) is as follows:
**Algorithm 1** AEDDPF**Require:**
observation data set $Q={\left\{q_{k}\right\}}_{k=1}^{N}$.**Ensure:**
membership matrix $B={\left[b_{rk}\right]}_{c\times N}$, clustering center $Z=\left\{z_{1},z_{2},\cdots ,z_{c}\right\}$.1:calculate the adaptive ellipse distance $dis_{k\ell }$ between any two data according to Equation (10);2:calculate the local density $\omega_{k}$ of any data point $q_{k}$ according to Equation (13);3:calculate the shortest distance $\varphi_{k}$ according to Equation (14);4:According to Steps 2 and Steps 3, obtain the density point set $H={\left\{\zeta_{k}\right\}}_{k=1}^{N}$ and draw a decision graph;5:Calculate the density mean point $\bar{\zeta }$ according to Equation (16) and Equation (17), and obtain the adaptive exponential function f(ω);6:According to Equation (18), the peak density point is determined and the corresponding data point is marked as the initial cluster center. The number of peak density points is recorded as *c*;7:Take fuzzy index m=2, initial iterations t=1, maximum iterations *T*, error threshold ε, initialized membership matrix $b^{\left(0\right)}={\left[b^{\left(0\right)}\right]}_{c\times N}$;8:Update the membership matrix and clustering center according to Equation (26)9:If $∥b^{\left(t\right)}-b^{\left(t-1\right)}∥\leq \varepsilon$, so the iteration ends;10:Otherwise t=t+1, repeat Step 8.

### 3.3. The Time Complexity Analysis of AEDDPF Clustering Algorithm

The characteristic of the AEDDPF algorithm is to use secondary clustering to correct the clustering results. First, the AEDDPF algorithm uses the improved density peak algorithm to perform the first clustering to obtain the initial cluster center point. The obtained initial cluster center points are very close to the actual optimal classification result. Secondly, the AEDDPF algorithm takes the initial clustering result as the input and uses the GK algorithm to perform secondary clustering and obtain more accurate clustering results. Although the AEDDPF algorithm uses a secondary clustering method to process the data, the cluster center point obtained by the AEDDPF algorithm during the primary clustering is closer to the final cluster center point, which reduces the number of iterations during the secondary clustering. Therefore, the time consumption of the algorithm is not very large, which can meet the real-time requirements of the system.

When clustering the data set, suppose the time complexity of the GK algorithm to be completed one iteration is $O(n_{1})$ and the total iteration number of the GK algorithm to process the data is *N*, so the total time complexity of completing the algorithm is $O(N*n_{1})$. Suppose the time complexity of the first clustering of AEDDPF algorithm is $O(n_{2})$ and the number of secondary clustering of AEDDPF algorithm is *M*, so the time complexity of AEDDPF can be obtained as $O\left(n_{2}\right)+M*O\left(n1\right)$. The improved density peak algorithm is used by the AEDDPF algorithm for the initial clustering to obtain the initial cluster center. The cluster centers obtained above are very close to the real cluster centers, which effectively reduces the iteration number in the secondary clustering of the AEDDPF algorithm. Therefore, the iteration number *M* of the AEDDPF algorithm in the secondary clustering is much smaller than the total iteration number *N* of the GK algorithm, and the condition M≪N is obtained. According to the above analysis, the time complexity of the AEDDPF algorithm satisfies $O\left(n_{2}\right)+M*O\left(n1\right)\ll O\left(n_{2}\right)+N*O\left(n_{1}\right)$. The time consumption of the AEDDPF algorithm is much smaller than the time consumption that is a simple superposition of the improved density peak algorithm and the GK algorithm. At the same time, the time consumption of the algorithm will be slightly greater than the improved peak density algorithm, but it can fully meet the real-time requirements of the system.

## 4. Experimental Results

### 4.1. Radar Data Processing

**Figure 4:**
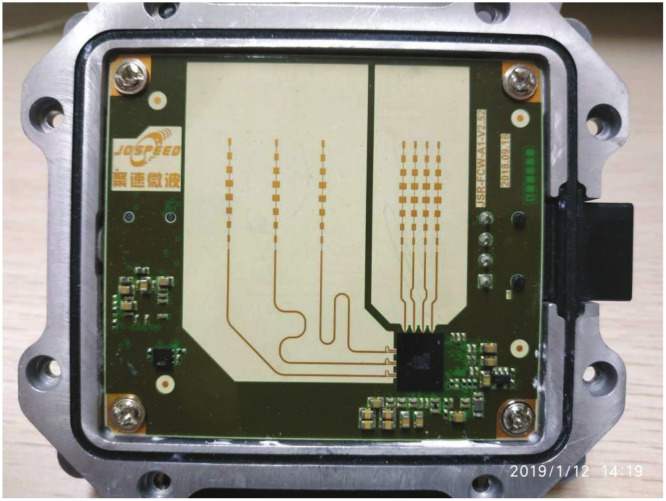
Multi-target FSK (Frequency-Shift Keying, FSK)radar.

The data used in this paper is obtained from the multi-target traffic radar, and the actual radar diagram is shown in Figure 4. The radar can be installed on the roadside or on the top of the road. The horizontal detection range of the radar is 3 to 5 lanes, and the vertical detection range of the radar is 50 to 300 meters. The multi-target traffic radar in this paper is based on the FSK (Frequency-Shift Keying, FSK) system and it has many advantages in the field of road traffic [30]. The FSK system radar can automatically shield targets whose radial velocity is zero relative to the radar. In the same way, stationary targets on the road such as railings, manhole covers, and green belts can be automatically shielded by the radar. The FSK system radar has a strong anti-interference ability and is usually used to detect motor vehicle targets on the road [31]. Through the above analysis, the AEDDPF algorithm will be studied based on the FSK system.

The radar system in this paper mainly includes radar, camera, and antennas. The radar transmits radar signals through two antennas and then receives reflected signals from vehicle targets. At the same time, the camera is responsible for capturing the image information of the corresponding vehicles on the road. The radar data processing flow is as follows:

- Step 1: Data acquisition: When the vehicle target enters the radar irradiation area, the radar antenna receives the reflected signal from the target.
- Step 2: Data preprocessing: The radar demodulates the echo signal. After that, noise removal and spectrum processing are performed on the demodulated signal to obtain a time-domain radar signal.
- Step 3: Radial distance acquisition: Use the radar ranging Equation (27) to obtain the radial distance between the vehicle target and the radar.
  (27) $$R=\frac{CT}{2\Delta F},$$
  where *R* is the radial distance between the radar and the target vehicle, *C* is the speed of light, *T* is the period of the transmitted signal, and ΔF is the frequency modulation bandwidth.
- Step 4: Direction angle acquisition: Use angle Equation (28) to calculate the angle between the vehicle’s driving direction and the radar beam direction.
  (28) $$\theta =\arcsin\left(\frac{\lambda \cdot \Delta \varphi_{12}}{2\pi \cdot \Delta d_{12}}\right),$$
  where θ is the angle between the driving direction of the vehicle and the direction of the radar beam, λ is the wavelength of the radar, $\Delta \varphi_{12}$ is the phase difference of the two received signals, $\Delta d_{12}$ is the distance between the two receiving antennas.
- Step 5: Coordinate conversion: The vertical distance from the radar installation position to the ground is known as *h*, and the two-dimensional coordinates q=(x,y) of the vehicle target in the radar coordinate system can be obtained using Equation (29).
  (29) $$\left\{\begin{array}{c} x=R\cdot \sin\theta \\ y=\sqrt{{\left(R\cdot \cos\theta \right)}^{2}-h^{2}}. \end{array}\right.$$

After the raw data is processed, the (R,θ) coordinate in the polar coordinate system is converted to the (x,y) coordinate in the rectangular coordinate system. The position coordinate (x,y) of the detection target in the two-dimensional form can intuitively display the position information of the detection point of the vehicle. The set of these position coordinate points is the radar dataset to be processed in this paper.

In the radar scenario of this paper, the size of the dataset is related to the number of vehicles. Generally, the number of radar reflection points for small cars in the scene is about 6-8, and the number of radar reflection points for large cars in the scene is about 10-15. When there are vehicles in the four lanes within the radar measurement range, there are about 60 points in the scene of this paper.

The measurement data of the radar in the actual scene is first collected and used as the training dataset. After that, the training dataset is used for parameter training calculation on the computer. After training the better parameters, the algorithm with the adjusted parameters is rewritten into the radar system installed above the road, and the system is tested in real-time to detect the relevant performance of the algorithm. If the algorithm runs poorly, the relevant dataset needs to be downloaded. After that, the downloaded data set is added to the original training data set to obtain a new training dataset. Based on the new training dataset, the computer re-trained and calculated the parameters of the algorithm, and the original parameters of the algorithm were further optimized. Under normal circumstances, the above process requires multiple cycles to obtain the optimal parameters.

**Figure 5:**
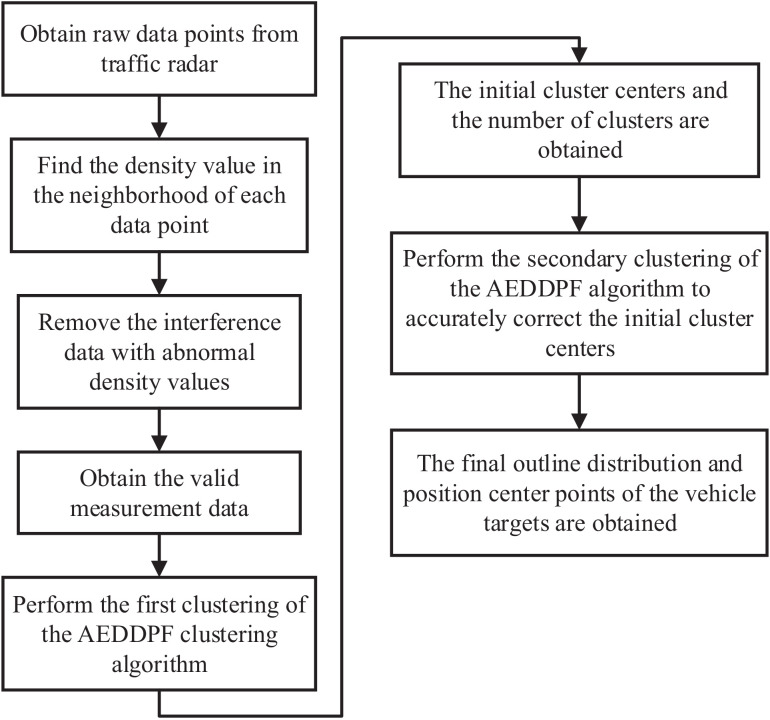
Flow chart of data processing.

FSK system radar has strong anti-noise interference ability. It can automatically shield targets whose radial velocity is zero relative to the radar. Therefore, the noise of stationary targets on the road such as railings, manhole covers, and green belts can be automatically filtered by the radar. In the four-lane highway scenario studied in this paper, there are only high-speed vehicle targets. At the same time, there are no pedestrians and other non-maneuverable targets in the above scene. Traffic jams occasionally occur in the scene. At this time, the speed of the vehicles in the scene is close to zero, and the FSK system radar will shield them. In this case, the algorithm will stop running. However, there may be mirror interference between adjacent vehicle targets measured in this paper. For mirror interference, we use density factors to filter out measurement points with abnormal speed and position. Therefore, before running the clustering algorithm, the radar mirror interference will be filtered out. In addition to the above interference, there is no other noise interference in the system. When comparing other methods, the same data processing flow is also used. The data processing of the proposed algorithm is shown in Figure 5.

### 4.2. Experimental Results and Comparison

**Figure 6:**
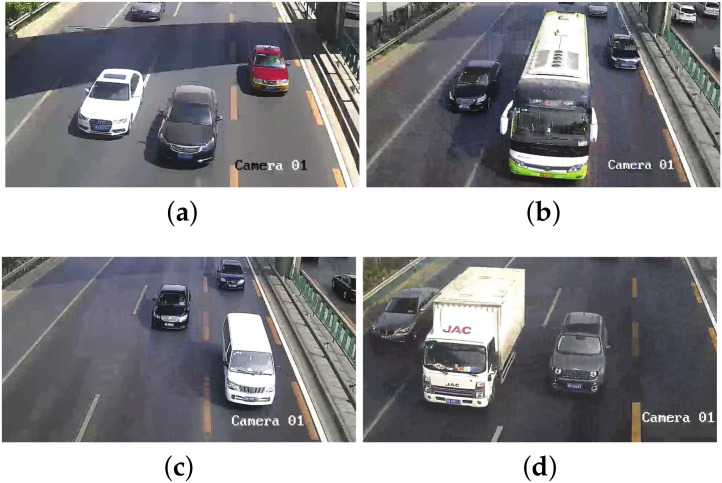
Four sets of real radar scenes in the experiment: (**a**) Scene1; (**b**) Scene2; (**c**) Scene3; (**d**) Scene4.

The experiment selected four sets of actual scene data obtained by multi-target traffic radar to explain the performance of the AEDDPF algorithm. In the actual scene, the vehicle is a large-sized target. When two vehicles are driving at a short distance in a horizontal or vertical direction, the radar will have a situation where the detection target points are not easy to distinguish. As shown in Figure 6, the four radar scenes are labeled Scene1, Scene2, Scene3, Scene4, respectively. In the four sets of real scenes, there are multiple vehicles with very close horizontal and vertical distances or driving side by side. In Scene1, the white car, and the black car travel side by side, and the driving distance is close, as shown in Figure 6a. In Scene2, the horizontal distance between the car in the second lane and the bus in the third lane is close, and the longitudinal distance between the bus and the black car in the fourth lane is close, as shown in Figure 6b. In Scene3, the longitudinal travel distance of the three vehicles is close, as shown in Figure 6c. In Scene4, the three vehicles are approaching laterally, as shown in Figure 6d.

**Figure 7:**
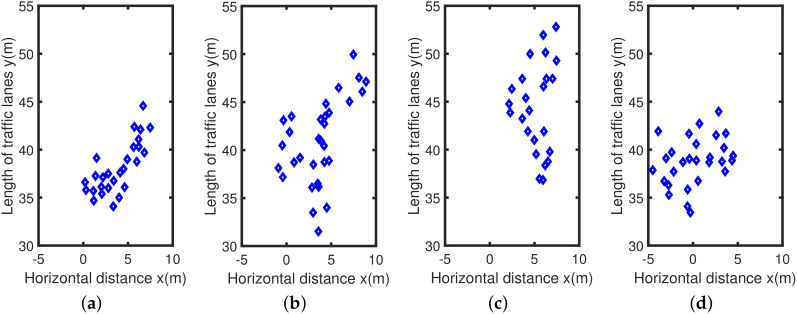
Two-dimensional radar dataset: (**a**) Dataset 1; (**b**) Dataset 2; (**c**) Dataset 3; (**d**) Dataset 4.

The actual detection chart is shown in Figure 6, and the total number of points in the radar data set is relatively small. Among them, each data set has about 20 to 30 detection points, corresponding to about 6 to 15 detection points for a car. The radar detects that the vehicle is a directional elliptical cluster, that is, the long half axis of the ellipse is parallel to the vertical direction of the coordinate axis, and the short half axis is parallel to the horizontal direction of the coordinate axis. The four radar detection maps in Figure 7 correspond to the four real vehicle scenes in Figure 6, where the horizontal axis represents the lateral distance of the lane *x*, and the vertical axis represents the length of the lane *y*, all in meters. The radar data collection method is one of the reasons for the above phenomenon. In a traffic scene, the vehicle target detected by the radar is not a point target, but a target with multiple scattering points. It is precise because of the discrete state of the target point that the horizontal and vertical distance between different point clusters in the actual detection data is small. Besides, the different sizes of the vehicle will also affect the effective points of the detected target. Corresponding to Figure 6b and Figure 7b, the bus body is long and it takes a long time to pass through the area to be measured, so there are many corresponding effective points. Similarly, the body of the car is short and the time to pass the area to be tested is short, so the corresponding detection points are few.

In this section, we analyze the AEDDPF algorithm through experiments and compare the proposed algorithm with DBSCAN, *k*-means, FCM, GK, and Euclid-ADDPF algorithms. To better prove the clustering effect of the AEDDPF algorithm, we replaced the adaptive elliptic distance part of the proposed AEDDPF algorithm with the adaptive Euclidean distance and obtained the adaptive Euclidean distance density peak fuzzy (Euclid-ADDPF) clustering algorithm. The Euclid-ADDPF algorithm will be used as the comparison algorithm of the AEDDPF algorithm for experiments.

The experiment mainly verifies the following three advantages of the AEDDPF algorithm:

- The AEDDPF algorithm can simply and adaptively select the initial cluster center, and the error between the center point after initialization and the real center point of the data is small.
- The AEDDPF algorithm can more accurately describe the elliptical structure of the data obtained by radar measurement vehicles and has a good clustering effect.
- Compared with other clustering algorithms, the AEDDPF algorithm has higher accuracy and better stability.

#### 4.2.1. The Initial Clustering Center Result of the AEDDPF Algorithm

To illustrate the effectiveness of the initial center selection, this paper compares the clustering centers after the AEDDPF algorithm initialization with the real data center points of four experimental scenarios. In Section 3.1.2, the AEDDPF algorithm calculates the density point set $H={\left\{\zeta_{k}\right\}}_{k=1}^{N}$ and obtains the decision graph by the element coordinates in the density point set *H*. After that, the average value of the horizontal and vertical coordinates of all elements in the density point set is calculated to obtain the density average point $\bar{\zeta }\left(\bar{\omega },\bar{\varphi }\right)$. The density mean point $\bar{\zeta }$ is brought into the adaptive exponential function Equation (17), and the adaptive exponential function expression is obtained.

**Figure 8:**
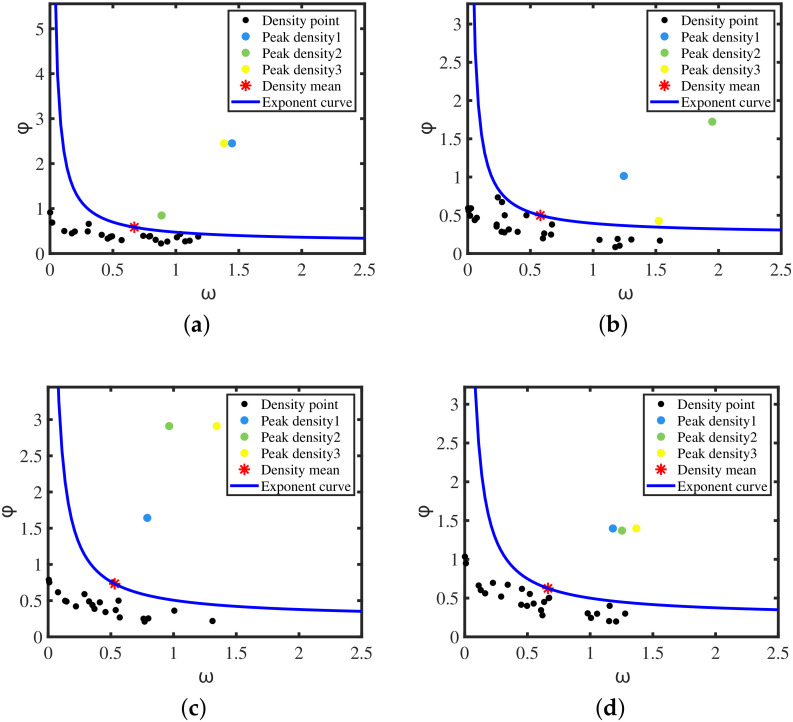
The decision chart corresponding to the radar detection data Dataset 1 to Dataset 4: (**a**) Decision graph of Dataset1; (**b**) Decision graph of Dataset2; (**c**) Decision graph of Dataset3; (**d**) Decision graph of Dataset4.

The experimental results are shown in Figure 8. The points in the data set are arranged in a decreasing density manner. The abscissa is the point density ω and the ordinate is the point distance φ. The red * is the mean density point $\bar{\zeta }\left(\bar{\omega },\bar{\varphi }\right)$. The blue curve is the adaptive exponential function, and the curve passes through the mean density point $\bar{\zeta }$. The color points are the initial cluster center points, which are the density peak points. The black points below the curve are the density point concentration except for the density peak point.

As shown in Figure 8, the colored points in the decision diagram are prominently distributed in the upper right part of the diagram and have large ω and φ values. These points have no data points with a higher density than they are in a larger neighborhood. These color points meet the two condition of the cluster center, which is the peak density point. The adaptive exponential function f(ω) divides the decision graph into two regions, and the colored point above the curve is used as the initial point of the cluster center.

The black density points below the curve f(ω) have three distributions in Figure 8:

- The density points have high ω values, but the φ values are very small, and they are all distributed in the lower right corner of the decision graph. This indicates that there are more dense points near their neighbors, which does not meet the criterions of the cluster center. These points are non-peak points.
- The ω values and φ values of density points are in the middle. This shows that there are more dense points near their neighbors, which does not meet the condition of cluster centers. Such points are also non-peak points.
- The density points have higher φ values, but the ω values are very low, all distributed in the upper left corner of the graph. This indicates that they are outlier non-peak points.

To show that the initial clustering center point obtained by the AEDDPF algorithm is closer to the real data center, this paper introduces distance error rate (DER) as the evaluation index of the initial clustering center, the expression is

(30) $$DER=\frac{d_{\mu }}{\sqrt{V^{2}+L^{2}}}\times 100\%.$$

where the center distance value $d_{\mu }$ represents the distance between the true center of the data set and the initial clustering center obtained by the algorithm, *V* and *L* are the horizontal distance and vertical distance of the data set, defined as

(31) $$\left\{\begin{array}{c} V=x_{\max}-x_{\min}\\ L=y_{\max}-y_{\min}, \end{array}\right.$$

where $x_{\max}$ and $x_{\min}$ respectively represent the maximum and minimum values of the abscissa of the points in the data set. $y_{\max}$ and $y_{\min}$ respectively represent the maximum and minimum values of the vertical coordinate of the points in the data set.

As shown in Table 1, the first column is the serial number of the four datasets. The second column is the cluster center number corresponding to the dataset. The third column is the true center coordinates of the four data sets.The true center coordinates (the true ground positions of the vehicle) are obtained by the lidar with high measurement accuracy. The data result obtained by the lidar measurement is used for the parameter calibration of the embedded algorithm, and the result is also used as a reference for the precise position of the target.The fourth column is the three initial cluster center coordinates obtained after the AEDDPF algorithm is run. The fifth column is the distance between the true center point and the initial cluster center. The sixth column is the DER of the initial cluster center. Table 1 shows that the number of clustering centers obtained by the algorithm initialization corresponds to the actual number of vehicle targets in Figure 7. This shows that the AEDDPF algorithm can select the correct number of clustering centers from the decision graph through the adaptive exponential function.The error values DER in Table 1 are less than 11%, which proves that the AEDDPF algorithm initializes the cluster center coordinates relatively close to the actual center coordinates of the radar datasets. The initial clustering center of the GK clustering algorithm is generally selected randomly. In comparison, the initial clustering center of the AEDDPF algorithm is obtained through the selection of the density peak point by the adaptive exponential curve. The experimental results also show that the obtained initial cluster center point is closer to the actual optimal classification result.

The selection process of the initial clustering center of the AEDDPF algorithm proposed in this experimental scenario is effective, and the selected initial clustering center point is ideal. The above experiments verify the effectiveness of the AEDDPF algorithm to initialize cluster centers in this paper.

#### 4.2.2. Comparison of Experimental Results

This part mainly compares and analyzes the running effects of DBSCAN, *k*-means, FCM, GK, Euclid-ADDPF, and AEDDPF algorithms in detail. For the DBSCAN algorithm, the neighborhood radius is ε=2, and the minimum number of points required to form a dense area is MinPts=5. For the *k*-means algorithm, the initial center number is taken as 3. For the FCM and GK algorithm, the initial center number is taken as 3 and the fuzzy index is taken as 3. For the Euclid-ADDPF algorithm, the fuzzy index of the algorithm is taken as 2. For the AEDDPF algorithm, the adjustment factor of the adaptive Euclidean distance is taken as α=1, and the fuzzy index is taken as m=2.

**Figure 9:**
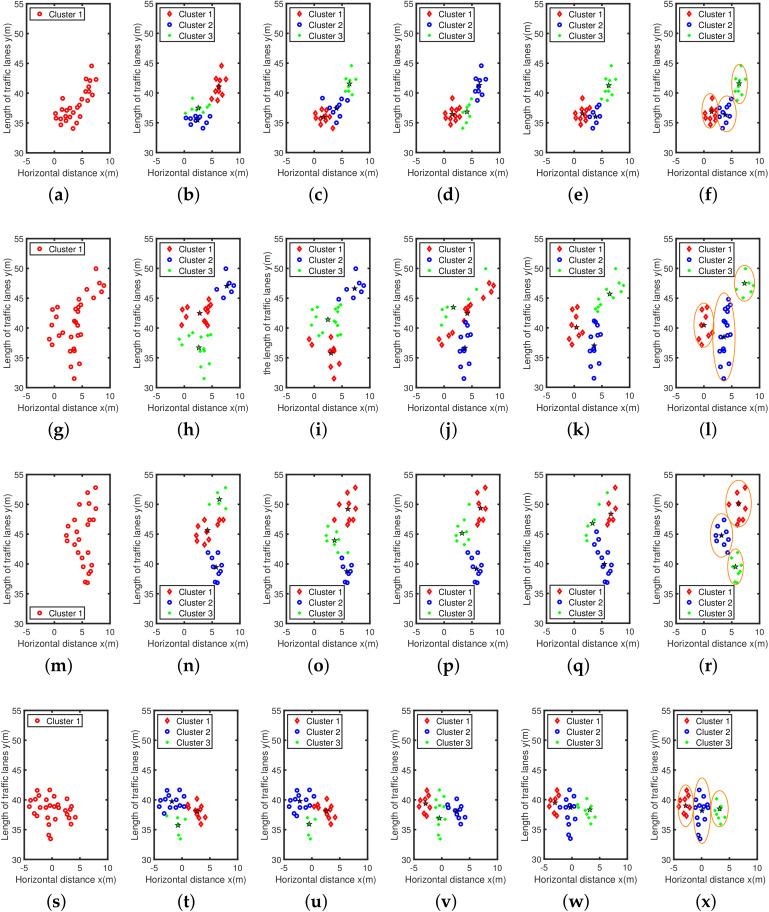
Clustering results of DBSCAN, *k*-means, FCM, GK, Euclid-ADDPF, and AEDDPF for 4 datasets: (**a**) DBSCAN Dataset1; (**b**) *k*-means Dataset1; (**c**) FCM Dataset1; (**d**) GK Dataset1; (**e**) Euclid-ADDPF Dataset1; (**f**) AEDDPF Dataset1; (**g**) DBSCAN Dataset2; (**h**) *k*-means Dataset2; (**i**) FCM Dataset2; (**j**) GK Dataset2; (**k**) Euclid-ADDPF Dataset2; (**l**) AEDDPF Dataset2; (**m**) DBSCAN Dataset3; (**n**) *k*-means Dataset3; (**o**) FCM Dataset3; (**p**) GK Dataset3; (**q**) Euclid-ADDPF Dataset3; (**r**) AEDDPF Dataset3; (**s**) DBSCAN Dataset4; (**t**) *k*-means Dataset4; (**u**) FCM Dataset4; (**v**) GK Dataset4; (**w**) Euclid-ADDPF Dataset4; (**x**) AEDDPF Dataset4.

Combining Figure 9 and Table 2, we can more fully understand the clustering effect of different algorithms in different radar scenarios.The analysis of the clustering effect of the four scenes is as follows:

- Scene1:The lateral distance of the two vehicles is close. Figure 9a shows that the DBSCAN algorithm cannot correctly classify Dataset1. As shown in Figure 9b, when the *k*-means algorithm clusters Dataset1, the shape of the blue and green clusters is wrong. The relative positions of the cluster center points of these two clusters are incorrect, which is inconsistent with the positions of the white and black cars in Scene1. As shown in Figure 9c, when the FCM algorithm clusters Dataset1, the shape of the blue and red clusters is wrong, and the relative positional deviation of the cluster center points of these two clusters is large. Figure 9d shows that the clustering effect of GK is better. As shown in Figure 9e, the Euclid-ADDPF algorithm has a better overall clustering effect, but the red and blue clusters have irregular shapes, which are somewhat different from the clustering effect of AEDDPF. As shown in Figure 9f, the AEDDPF algorithm has the best clustering effect on Dataset1, and can completely describe the elliptical shape of point clusters. As shown in the Dataset1 column of the Table 2, the comprehensive DER of the three clustering centers obtained by the AEDDPF algorithm is the smallest, and the DER values are all less than 2%.
- Scene2: The driving distance of the big car and the two small cars is close. Figure 9g shows that the DBSCAN algorithm cannot correctly classify Dataset2. As shown in Figure 9h, when the *k*-means algorithm clusters Dataset2, the direction of the green and red clusters is horizontal, and the cluster center position of the two clusters does not match the position of the two vehicles in the actual scene. As shown in Figure 9i, when the FCM algorithm clusters Dataset2, the direction of the green and red clusters is horizontal, and the cluster center position of the two clusters does not match the position of the two vehicles in the actual scene. It can be seen from Figure 9j that GK has obtained elliptical clusters, but the red clusters and green clusters are oblique, rather than parallel to the longitudinal axis, so the direction of the clusters is wrong. As shown in Figure 9k, the Euclid-ADDPF algorithm can cluster the correct number of clusters. Among them, the shape of the green cluster does not conform to the real shape of the car, and the longitudinal length of the blue cluster does not match the real length of the bus in the real scene. As shown in Figure 9l, the AEDDPF algorithm clusters Dataset2 into three ellipse-shaped clusters, and the cluster center position of the three clusters is in accordance with the actual situation. In the Dataset2 column of Table 2, the combined DER of the AEDDPF algorithm is the smallest, and the DER values are all less than 1%.
- Scene3: The three cars are close to each other in the longitudinal distance. Figure 9m shows that the DBSCAN algorithm cannot correctly classify Dataset 3. As shown in Figure 9n–Figure 9q, the *k*-means, FCM, GK, Euclid-ADDPF, and AEDDPF algorithms can correctly cluster the position, orientation, and shape of clusters in Dataset3. In comparison, the AEDDPF algorithm is more reasonable for clustering edge points of clusters. From the Dataset3 column in Table 2, under the condition that the clustering effects of *k*-means, FCM, GK, and AEDDPF are similar, the DER of the AEDDPF algorithm is generally ideal, and the DER values are all less than 2%.
- Scene4: The three cars are close to each other in the lateral distance. Figure 9s shows that the DBSCAN algorithm cannot correctly classify Dataset4. As shown in Figure 9t, when the *k*-means algorithm clusters Dataset4, the green and blue clusters are in the wrong direction. Besides, the location of the cluster center point of the two clusters is wrong, which does not match the position of the white and gray cars in Scene4. As shown in Figure 9u, when the FCM algorithm describes Dataset4, the clustering center points of the green and red clusters are in the wrong position, which is inconsistent with the white and gray car positions in the Scene4 scene.It can be seen from Figure 9v that the clustering effect of GK algorithm is better. As shown in Figure 9w, Euclid-ADDPF clustered the correct number of clusters, but the shapes of the green and red clusters are somewhat different from the shape of the elongated ellipse of the car. As shown in Figure 9x, the three clusters of red, blue, and green clustered by the AEDDPF algorithm are all ellipses whose long semi-axis is parallel to the vertical axis, and the position of the cluster center of the three clusters is consistent with the Scene4. The Dataset4 column of Table 2 shows that the DER of the three central points of the AEDDPF algorithm is more ideal than other algorithms.

From the cluster analysis of Scene1 to Scene4, the AEDDPF algorithm ensures that the cluster center error rate is relatively small under the premise of ensuring the correct number of clusters in the cluster. Also, the AEDDPF algorithm can accurately describe the elliptical shape of the clusters in the datasets, and the clustering effect is better than the DBSCAN, *k*-means, FCM, GK, and Euclid-ADDPF algorithms.

**Figure 10:**
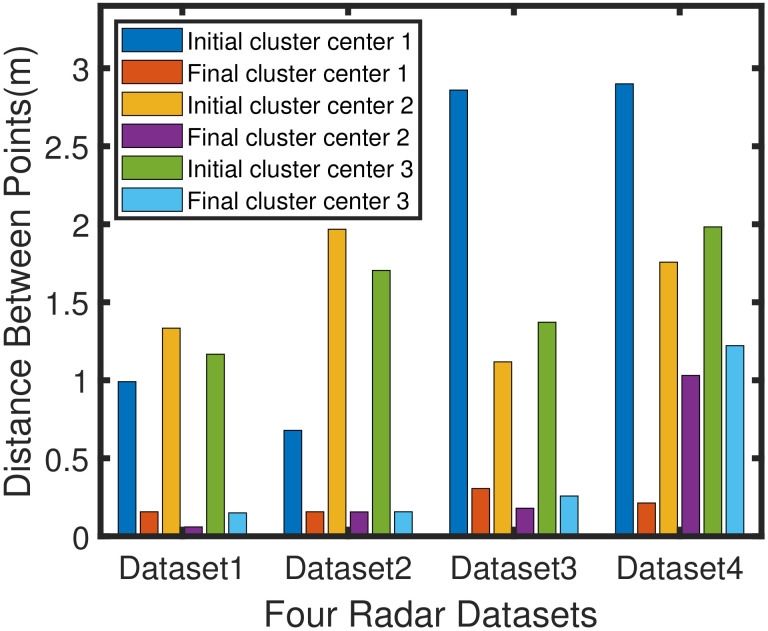
Adaptive ellipse distance density peak fuzzy (AEDDPF) algorithm initial cluster center point distance and final cluster center distance histogram comparison chart.

The initial clustering center point of the AEDDPF algorithm is used as the input data of the algorithm, so that the error distance of the final clustering center is reduced. In Figure 10, the initial error value is the fifth column of Table 1, and the final clustering result error distance value is the data corresponding to the Euclid-ADDPF row of the Table 2. The two sets of data are compared to form a histogram. In dataset 1, the final error distances of the three center points are lower than the initial error distance by 0.834m, 1.274m, and 1.017m, respectively. In dataset 2, the final error distances of the three center points are lower than the initial error distance by 0.522m, 1.812m, and 1.547m, respectively. In dataset 3, the final error distances of the three center points are lower than the initial error distance by 2.554m, 0.938m, and 1.114m respectively. In dataset 5, the final error distances of the three center points are lower than the initial error distance by 2.687m, 0.726m, and 0.761m, respectively.

To further illustrate the clustering performance of the algorithm, the accuracy rate (AR), running time, and the number of iterations of the five algorithms in four groups of scenarios can be compared. The clustering accuracy AR of the dataset is defined as follows:

(32) $$AR=\frac{DT}{DT+DF}\times 100\%,$$

where DT represents the number of correctly classified points and DF represents the number of incorrectly classified points.

**Figure 11:**
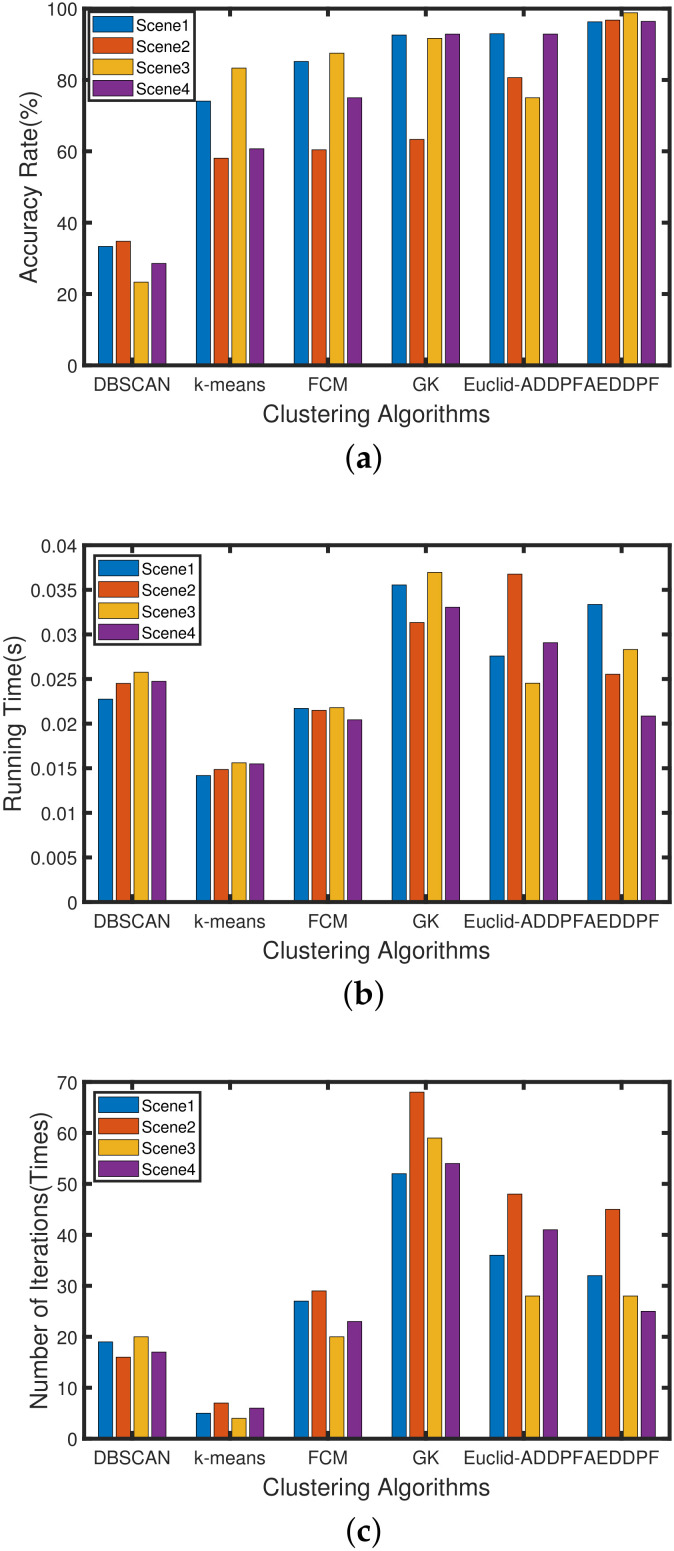
Histogram comparison of clustering results of different clustering algorithms in four radar scenes: (**a**) Average Accuracy (%); (**b**) Average Running Time (s); (**c**) Average Number of Iterations (*Times*).

Perform the same experiment for the six algorithms mentioned in this article. The experiment selects the above four traffic scenes to run 1000 times, and the average performance data of different algorithms is obtained. As shown in Figure 11a, the average accuracy values of the AEDDPF algorithm is the highest, and the accuracy of the algorithm is all above 95%. In particular, compared with the GK algorithm, the average accuracy of the AEDDPF algorithm in the 4 datasets is increased by 3.7%, 33.44%, 7.17%, and 3.57%, respectively. Compared with Euclid-ADDPF algorithm, the average accuracy rate in the 4 datasets of AEDDPF algorithm is increased by 3.34%, 16.12%, 23.83%, and 3.56%, respectively. As shown in Figure 11b, from Scene 1 to Scene 4, the average running time of the AEDDPF algorithm is in the middle position. Compared with the longest running GK algorithm, the average running time of the AEDDPF algorithm is reduced by 0.00219s, 0.005798s, 0.008641s and 0.012195s. As shown in Figure 11c, from scene 1 to scene 4, the average number of iterations of the AEDDPF algorithm is also in the middle position.

Through experiments and analysis, the proposed AEDDPF algorithm has the best clustering effect compared to DBSCAN, *k*-means, FCM, GK, and Euclid-ADDPF. At the same time, the running time and number of iterations of the proposed algorithm are also in the middle position. In order to compare the performance of each algorithm more comprehensively, the same experiment was performed for the six algorithms mentioned in this article. The experiment selected 10 groups of different traffic scenes to run 1000 times to obtain the comprehensive average data of different algorithms. The Table 3 shows that the average accuracy of the AEDDPF algorithm is as high as 97.52%. The average running time of the AEDDPF algorithm is in the middle. The average iterations number of the AEDDPF algorithm is about 26 times less than the GK algorithm. The clustering effect of DBSCAN and *k*-means in the current vehicle scene is poor, and their average accuracy rate is less than 70%. After comprehensively considering the above clustering indicators, the AEDDPF algorithm performs better in the current radar scenes than other algorithms.

**Figure 12:**
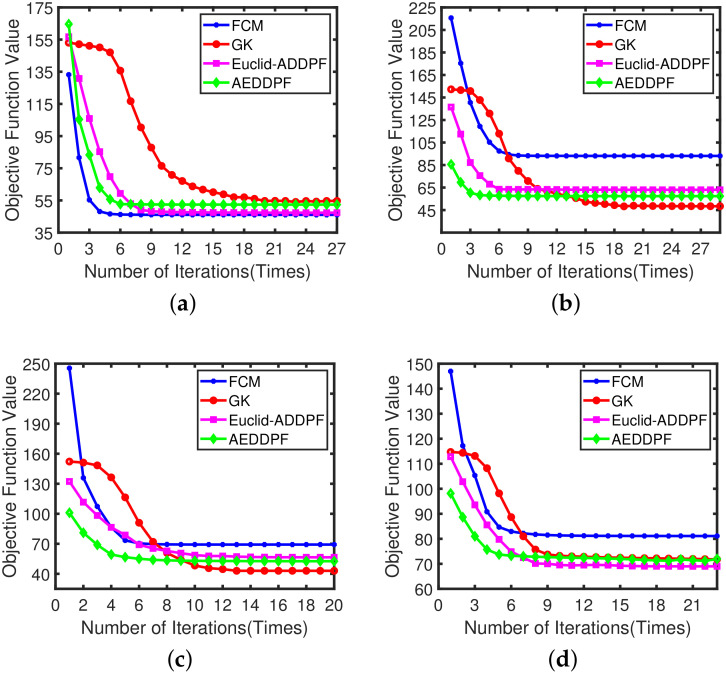
Decibels of fuzzy *c*-means (FCM), Gustafson-Kessel (GK), Euclidean distance density peak fuzzy (Euclid-ADDPF), and AEDDPF clustering algorithms were applied to the comparative experiment of Scene1 to Scene4 analyze the broken line graph: (**a**) Operational efficiency of Dataset1; (**b**) Operational efficiency of Dataset2; (**c**) Operational efficiency of Dataset3; (**d**) Operational efficiency of Dataset4.

The FCM, GK, Euclid-ADDPF, and AEDDPF algorithms are all fuzzy clustering algorithms, and the clustering accuracy is higher in the current experiment. The FCM, GK, Euclid-ADDPF, and AEDDPF algorithms are used for further analysis. Figure 12 shows the change of the convergence rate of the objective function with the number of iterations on the data sets corresponding to the four scenarios in this paper by the three algorithms. As the number of iterations increases, the objective function gradually decays. The decay rate is fast at the beginning. After a certain number of iterations, however, the decay rate becomes slower. When the objective function converges faster as the number of iterations increases, the algorithm is more efficient in clustering. In Figure 12, the AEDDPF algorithm’s curve stationary point appears relatively quickly. As shown in the four figures from Figure 12a to Figure 12d, the number of iterations of the position where the stationary point appears in the AEDDPF algorithm is reduced by 14, 15, 5, and 4 times compared with the GK algorithm, and compared to Euclid-ADDPF algorithm reduces 2 times, 2 times, 4 times and 2 times respectively.In other words, the overall performance of AEDPPF algorithm’s convergence speed is better.

In summary, the AEDDPF algorithm initializes accurately to obtain the coordinates and number of initial cluster center points. The effective initialization also reduces the number of iterations of the algorithm in the subsequent clustering process and improves the clustering accuracy. The above experiments show that the AEDDPF algorithm solves the problem that many traditional algorithms need to determine the number of clusters in advance. Besides, the algorithm has high average accuracy and a good clustering effect in the current radar scenes.

### 4.3. Discussion

The proposed AEDDPF algorithm can be applied to the multi-target radar in this paper. At the same time, the algorithm can also be applied to other radars such as lidar and high-precision radar. The multi-target radar in this paper is the FSK system. It can automatically shield stationary target interference. When other systems of radar (such as FCMW system radar) use this algorithm, the interference of stationary targets to the algorithm is needed to be removed in advance and the moving targets are extracted as measurement data. After that, the algorithm parameters need to be optimized and adjusted. In the radar scene data of this paper, the outline of the car is characterized by an elongated ellipse. The AEDDPF algorithm has a good clustering effect on the measurement data with a slender ellipse profile similar to the vehicle target. However, the clustering effect on the measurement data of the non-slender ellipse profile feature is average. For different types of data (such as image data), if the data does not meet the elliptical slender profile condition, this method is not applicable.

## 5. Conclusions

This paper solves the problem of low clustering accuracy of commonly used algorithms when vehicles travel at close range in a multi-target traffic radar scenario. Combining with previous thoughts, an adaptive ellipse distance density peak fuzzy clustering algorithm was proposed. There are two main ideas of the proposed AEDDPF algorithm:

- In order to obtain the initial cluster center coordinate value and the number of cluster centers, adaptive ellipse distance is used to accurately describe the structure of radar scene data and an exponential function is introduced to adaptively select cluster centers. This improvement improves the accuracy of the initial cluster center selection.
- The initial clustering center point is used as the subsequent input condition of the algorithm, which not only reduces the number of clustering iterations of the algorithm, but also makes the final clustering result of the algorithm more accurate.

In the experiment, real radar datasets are used to compare the clustering effect of the AEDDPF algorithm with DBSCAN, *k*-means, FCM, GK, and Euclid-ADDPF algorithms. Experimental results show that in the scenario of multi-target traffic radar driving vehicles at close range, the AEDDPF algorithm can cluster data points more efficiently. In the follow-up work, the AEDDPF algorithm may be used in engineering applications such as traffic radar target detection and tracking.

## Figures and Tables

**Table 1:** Comparison of the results of the initial cluster center and the true center of the datasets.

Dataset	Serial Number	True Center Coordinates	Initial Cluster Center Coordinates	Dis(m)	DER(%)
Dataset1	1	(1.498 36.963)	(2.015 36.118)	0.991	7.76
	2	(3.828 36.313)	(4.121 37.614)	1.334	10.44
	3	(6.221 41.467)	(6.200 40.300)	1.167	9.14
Dataset2	1	(0.201 40.372)	(−0.469 40.483)	0.679	3.25
	2	(3.554 38.471)	(3.503 36.504)	1.968	9.43
	3	(7.211 47.407)	(8.892 47.129)	1.704	8.16
Dataset3	1	(6.506 50.242)	(6.221 50.131)	2.86	1.82
	2	(3.266 44.632)	(2.157 44.775)	1.118	6.66
	3	(5.566 39.756)	(5.177 39.523)	1.372	2.70
Dataset4	1	(−2.752 39.198)	(−2.168 37.298)	2.9	10.37
	2	(1.103 38.147)	(−0.406 39.047)	1.757	8.70
	3	(3.172 37.285)	(4.356 38.876)	1.983	10.34

**Table 2:** Cluster center point coordinates.

Algorithm	Serial Number	Dataset1			Dataset2			Dataset3			Dataset4		
		Cluster Center Coordinates	dis (m)	DER (%)	Cluster Center Coordinates	dis (m)	DER (%)	Cluster Center Coordinates	dis (m)	DER (%)	Cluster Center Coordinates	dis (m)	DER (%)
k-means	1	(2.136 37.054)	0.644	5.04	(2.768 42.473)	3.317	15.89	(6.309 50.831)	0.621	3.69	(−1.811 39.733)	1.082	7.98
	2	(3.002 35.207)	1.38	10.81	(2.613 36.703)	2.002	9.59	(4.193 45.645)	1.373	8.17	(−0.732 35.737)	3.029	22.34
	3	(6.199 41.047)	0.421	3.30	(7.632 47.041)	0.558	2.67	(5.706 39.449)	0.337	2.01	(2.740 38.105)	0.927	6.84
FCM	1	(1.636 35.959)	1.013	7.93	(2.459 41.404)	2.483	11.89	(6.304 49.974)	0.336	2.00	(−2.178 39.789)	0.824	6.07
	2	(3.990 37.417)	1.116	8.74	(3.031 35.781)	2.74	13.13	(3.565 44.726)	0.313	1.86	(−0.487 35.942)	2.718	20.05
	3	(6.331 41.545)	0.135	1.06	(7.264 46.621)	0.788	3.77	(5.891 38.915)	0.902	5.36	(2.664 38.137)	0.992	7.32
GK	1	(1.697 35.626)	1.352	10.59	(0.166 40.232)	0.144	0.69	(6.550 49.360)	0.883	5.25	(−2.910 39.365)	0.23	1.69
	2	(3.170 37.434)	1.3	10.18	(3.875 38.937)	0.566	2.71	(3.221 45.164)	0.534	3.18	(−0.423 36.913)	1.962	14.47
	3	(6.250 41.242)	0.227	1.78	(7.603 46.798)	0.725	3.47	(5.817 39.178)	0.63	3.75	(2.735 38.065)	0.894	6.60
Euclid- ADDPF	1	(1.487 36.552)	0.411	3.22	(0.412 40.083)	0.358	1.71	(6.617 48.378)	1.867	11.11	(−3.056 39.482)	0.416	3.07
	2	(3.839 36.024)	0.289	2.26	(3.631 37.058)	1.415	6.78	(3.301 46.799)	2.167	12.89	(−0.273 38.682)	1.476	10.89
	3	(6.222 41.278)	0.189	1.48	(6.441 45.726)	1.85	8.86	(5.438 39.942)	0.226	1.34	(3.226 38.306)	1.022	7.54
**AEDDPF**	**1**	**(1.387 36.852)**	**0.157**	**1.23**	**(0.312 40.483)**	**0.156**	**0.75**	**(6.221 50.131)**	**0.306**	**1.82**	**(−2.856 39.012)**	**0.213**	**1.57**
	**2**	**(3.839 36.324)**	**0.016**	**0.13**	**(3.665 38.581)**	**0.155**	**0.75**	**(3.157 44.775)**	**0.179**	**1.07**	**(0.073 38.182)**	**1.03**	**7.60**
	**3**	**(6.322 41.578)**	**0.15**	**1.17**	**(7.321 47.519)**	**0.157**	**0.75**	**(5.677 39.523)**	**0.258**	**1.53**	**(3.226 38.506)**	**1.221**	**9.01**

**Table 3:** Comparison experiment of AEDDPF algorithm with other algorithms in radar dataset.

Algorithm	Evaluation Index		
	Average Number of Iterations (Times)	Average Accuracy (%)	Average Running Time (s)
DBSCAN	18	30.21	0.024621
*k*-means	5	68.49	0.014962
FCM	26	78.11	0.021353
GK	59	85.64	0.034219
Euclid-ADDPF	38	88.59	0.029485
**AEDDPF**	**33**	**97.52**	**0.027013**
